# Sex Differences in Remote Contextual Fear Generalization in Mice

**DOI:** 10.3389/fnbeh.2019.00056

**Published:** 2019-03-22

**Authors:** Arun Asok, Joud Hijazi, Lucas R. Harvey, Stylianos Kosmidis, Eric R. Kandel, Joseph B. Rayman

**Affiliations:** ^1^Department of Neuroscience, Jerome L. Greene Science Center, Columbia University, New York, NY, United States; ^2^Zuckerman Mind Brain Behavior Institute, Columbia University, New York, NY, United States; ^3^Howard Hughes Medical Institute, Columbia University, New York, NY, United States; ^4^Kavli Institute for Brain Science, Columbia University, New York, NY, United States

**Keywords:** fear generalization, remote generalization, contextual fear conditioning, fear memory, sex differences

## Abstract

The generalization of fear is adaptive in that it allows an animal to respond appropriately to novel threats that are not identical to previous experiences. In contrast, the overgeneralization of fear is maladaptive and is a hallmark of post-traumatic stress disorder (PTSD), a psychiatric illness that is characterized by chronic symptomatology and a higher incidence in women compared to men. Therefore, understanding the neural basis of fear generalization at remote time-points in female animals is of particular translational relevance. However, our understanding of the neurobiology of fear generalization is largely restricted to studies employing male mice and focusing on recent time-points (i.e., within 24–48 h following conditioning). To address these limitations, we examined how male and female mice generalize contextual fear at remote time intervals (i.e., 3 weeks after conditioning). In agreement with earlier studies of fear generalization at proximal time-points, we find that the test order of training and generalization contexts is a critical determinant of generalization and context discrimination, particularly for female mice. However, tactile elements that are present during fear conditioning are more salient for male mice. Our study highlights long-term sex differences in defensive behavior between male and female mice and may provide insight into sex differences in the processing and retrieval of remote fear memory observed in humans.

## Introduction

Post-traumatic stress disorder (PTSD) is a debilitating psychiatric illness that emerges following exposure to a life-threatening experience and is characterized by four symptom clusters: re-experiencing, avoidance, negative alterations in mood or cognition, and hyperarousal (APA, 2013). An important clinical manifestation of PTSD is the overgeneralization of fear or enhanced distress to environmental cues that resemble the life-threatening experience (APA, 2013). Patients who suffer from PTSD have greater difficulty in suppressing fear in a safe environment or in the presence of safety cues (Jovanovic et al., 2010). For example, in a laboratory setting, individuals with PTSD have greater difficulty relative to control subjects in discerning perceptually similar rings from those paired with a shock (i.e., fear-conditioned rings; Kaczkurkin et al., 2017). PTSD is therefore associated with broader generalization gradients (Grillon et al., 2009; Jovanovic et al., 2012; Homan et al., 2019; Starita et al., 2019).

The incidence of PTSD is significantly higher in women than in men (Kessler et al., 2005; Tolin and Foa, 2006). Given that PTSD involves alterations in fear learning and memory (Ross et al., 2017), understanding the environmental constraints that control fear generalization between sexes is an important area of research (Lissek et al., 2010; Dunsmoor and Paz, 2015; Lissek and van Meurs, 2015; Liberzon and Ressler, 2016; Lopresto et al., 2016; Jasnow et al., 2017). In this regard, rodent fear conditioning paradigms represent a powerful tool for examining how environmental parameters interact to influence fear generalization as a function of sex (Parsons and Ressler, 2013; Maeng and Milad, 2017; Asok et al., 2019a).

Like humans, when rodents are confronted with a potentially threatening stimulus or an environment, they must select an appropriate defensive response (Fanselow and Lester, 1988; Blanchard and Blanchard, 1989; Mobbs et al., 2015). Because current and past experiences are not identical, the selection of a response is based on the immediately available cues and contextual information that predict danger or safety. This process often entails the generalization of a defensive response (e.g., freezing in rodents) to an environment that was never explicitly learned to be dangerous (Dunsmoor and Paz, 2015; Dymond et al., 2015; Jasnow et al., 2017). In recent years, rodent behavioral studies have discovered a variety of molecular, cellular, and neural circuit mechanisms that influence fear generalization (for review, see Asok et al., 2019a). These studies have revealed how internal states may interact with environmental contingencies to modulate sex differences in fear generalization (Day et al., 2016; Keiser et al., 2017). For example, ovariectomized female rats given estradiol replacement exhibit enhanced fear generalization *via* activation of cytosolic estrogen receptors in the hippocampus (Lynch et al., 2013, 2016). However, the role of female hormonal fluctuations in fear generalization is not so clear in that other studies have shown that hormonal changes: (1) may have a greater influence on fear extinction (Milad et al., 2009); and (2) do not influence contextual fear generalization (Keiser et al., 2017).

Despite controversy on the role of hormones in fear acquisition, fear extinction, or fear generalization, these studies have been critical for probing the biological factors that influence aversive experiences in females. Yet, much of this work has focused on fear generalization at recent time points after conditioning (i.e., 24–48 h). Given that PTSD is associated with progressive and chronic symptomatology as well as a higher incidence in women (Kessler et al., 2005; Nemeroff et al., 2006; Tolin and Foa, 2006), it is therefore important to examine the environmental factors which modulate generalization between sexes over longer time intervals, as this may identify key environmental variables that modulate sex-dependent fear generalization in PTSD.

Environmental, or contextual, elements exert a powerful influence over fear generalization. This is especially true for rodent studies that examine fear generalization using a contextual fear conditioning (CFC) paradigm, where the elements of an environment (e.g., sounds, lighting, textures, space, etc.) are bound into a unitary contextual representation (O’Reilly and Rudy, 2001; Rudy et al., 2004; Rudy, 2009; Maren et al., 2013). In CFC, a neutral stimulus (i.e., a unique context) is paired with an unconditioned stimulus (US) such as a foot shock—which has been suggested to serve as a proxy for trauma (Liberzon and Ressler, 2016). The neutral stimulus is associatively transformed into a conditioned stimulus (CS) that can subsequently elicit freezing on future presentations (Maren, 2001). Following CFC, generalization occurs when a context that is perceptually related, but not identical, to the conditioning context elicits a similar conditioned response (Asok et al., 2019a). Moreover, the saliency of particular stimulus elements during conditioning can have a profound influence over whether fear becomes generalized. For example, tactile feedback from the electrified grid floors as well as odors present in the conditioning chamber are particularly salient features for mice and are capable of modulating fear memory, generalization, and context discrimination (Huckleberry et al., 2016).

In addition, fear generalization is subject to a number of temporal constraints. The order of context exposure prior to, or following conditioning, as well as the similarity between the conditioning and testing contexts, can produce differential effects on fear generalization (Tronel et al., 2005; Huckleberry et al., 2016; Keiser et al., 2017). However, fear generalization can naturally emerge with the passage of time in both rodents (Wiltgen and Silva, 2007) and humans (Leer et al., 2018), and may accompany the normal systems consolidation of a fear memory (Biedenkapp and Rudy, 2007; Wiltgen et al., 2010; Dudai et al., 2015; Poulos et al., 2016).

In light of the sex-dependent, contextual, and temporal factors that influence fear generalization, we examined how pre- and post-conditioning exposure to different contexts and elements of a context influence fear generalization and context discrimination at remote time intervals in both male and female mice. Our study highlights several key environmental parameters that may contribute to the stress-related, sex-dependent emergence of fear generalization.

## Materials and Methods

### Animals

Wild-type male and female mice (C57BL/6J background) were obtained from Jackson Laboratory (Bar Harbor, ME, USA) at 9–10 weeks of age. Animals were housed in the vivarium at the Zuckerman Institute at Columbia University, and maintained on a standard 12 h : 12 h light-dark cycle with *ad libitum* access to food and water. This study was carried out in accordance with the recommendations of the Animal Research Handbook made available by the Office of the Executive Vice President for Research at Columbia University. The protocol was approved by the Institutional Animal Care and Use Committee (IACUC) at Columbia University.

### Behavioral Experiments

#### Estrous Cycle

Naturally cycling females were used in all experiments, given that the C57BL/6J background is relatively insulated from the effects of the estrous cycle with respect to fear conditioning (Meziane et al., 2007; Keiser et al., 2017). Indeed, the effects of estrous phase on fear memory in rodents are more relevant to fear extinction (Milad et al., 2009; Blume et al., 2017). Nevertheless, in our study, we performed limited visual monitoring of estrous cycle phase, as described in Byers et al. (2012). Among five female cohorts evaluated in remote generalization experiments and for which estrous phase was assessed (*n* = 75 mice in total), there were no significant differences in phase distribution between experimental groups on the day of fear conditioning ($\chi_{\left(8\right)}^{2}$ = 8.73, *p* = 0.3656). The percentage of mice in proestrus—when estradiol and progesterone levels are highest—on the day of fear conditioning ranged from 0 to 20% (0–3 mice per group, for a total of six animals in proestrus). Combining these five groups (*n* = 75 mice in total) and measuring post-shock freezing levels as a function of estrous phase, we detected no significant differences between groups by one-way analysis of variance (ANOVA; *F*_(2,72)_ = 0.2827, *p* = 0.7546). Although potential effects of proestrus on retrieval testing cannot be entirely ruled out due to the relatively small representation of proestrus mice during training, exclusion of these animals from statistical analyses of behavioral data has no impact on significance or interpretation (data not shown), and so these data points were retained. Our results are consistent with those of earlier reports (Meziane et al., 2007; Keiser et al., 2017).

### Contextual Fear Conditioning and Generalization

All behavioral experiments were conducted on mice between 12–14 weeks of age. Fear conditioning experiments were conducted using a cubic chamber with the following dimensions: 30 cm (L) × 24 cm (W) × 21 cm (H). The fear conditioning chamber was housed in a sound-attenuating enclosure equipped with an infrared camera for automated measurement of freezing, which was quantitated using Video Freeze software (Med Associates, Inc., St Albans City, VT, USA). For standard CFC, mice were exposed to a 3 min session, with a 2 s shock (0.7 mA) presented at 2 min and again at 2.5 min. In the brief training protocol, two shocks of the same intensity and duration as in the standard protocol were administered over the course of an 8 s session, whereupon animals were immediately returned to their home cages. Control animals were exposed to the fear conditioning chamber for 3 min in the absence of shock. Three contexts were used as indicated: Context A (70% ethanol odorant, white light, metal floor grid, no roof insert); Context B (4% peppermint extract, no light, smooth flooring, triangular roof insert); and Context C (same as Context B, but with metal floor grid used in Context A). Animals were only exposed to foot shocks during initial fear conditioning in Context A. To evaluate retrieval, animals were exposed to the indicated test contexts for a duration of 3 min. For context pre-exposure experiments, test mice were placed in Context A for 10 min in the absence of foot shocks on one or two consecutive days as indicated, and then subjected to standard fear conditioning in Context A the following day. Control mice in the pre-exposure experiments were not pre-exposed to Context A, but were subjected to the same course of fear conditioning and retrieval as the other test groups. At the conclusion of the fear conditioning or retrieval sessions, mice were immediately returned to their home cages. Contextual memory retrieval or generalization were evaluated at 24 h, 48 h, or 21 days and 22 days later, as specified, in the absence of shocks. For quantitation of all behavioral data from fear conditioning and generalization experiments, % time freezing was used.

### Data Analysis

All data were analyzed using the total percentage of time spent freezing and by computing a discrimination index [% time freezing in Context A/(% time freezing in Context A + Context B)]; (Wiltgen and Silva, 2007). All values are reported as the mean ± standard error of the mean (SEM). All behavioral data were analyzed by one-, two-, three-way ANOVA, or *t*-test as specified, using GraphPad Prism 7 software (GraphPad Software, Inc.). Cohort and sample sizes are specified in the text and figures. *Post hoc* comparisons performed after significant ANOVA results are specified when used. Statistical significance was set at *p* < 0.05 “*”, *p* < 0.01 “**”, *p* < 0.001 “***”, and *p* < 0.0001 “#”.

## Results

### Experiment 1: Remote Contextual Fear Generalization in a Distinct Context Is Modulated by Sex and Test Order

**Figure 1:**
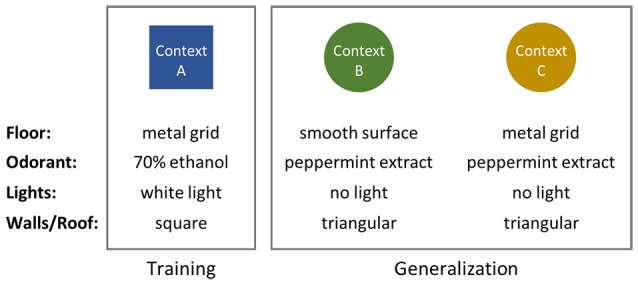
Schematic of contexts used in fear conditioning and generalization experiments. Context B was designed to be as perceptually distinct as possible from the training context (Context A). Context C retains the metal floor grid used in Context A, but is otherwise identical to Context B.

**Figure 2:**
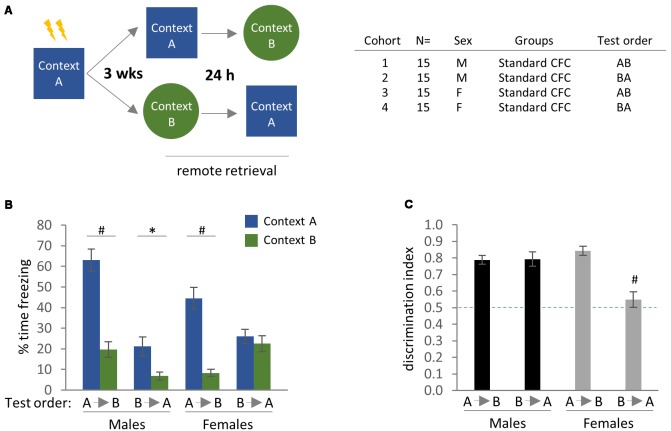
Remote contextual fear memory and generalization with perceptually distinct training and generalization contexts (Contexts A and B, respectively). **(A)** Experimental design and cohort information. **(B)** Effect of test order on freezing behavior in the training context (Context A) vs. a distinct novel context (Context B) at 3 weeks after standard contextual fear conditioning (CFC). Bonferroni *post hoc* comparisons following three-way analysis of variance (ANOVA) are indicated. **(C)** Discrimination index, calculated as % Freezing in Context A/(% Freezing in Context A + % Freezing in Context B). ^#^*p* < 0.0001 for effect of test order in females, Bonferroni *post hoc* test following two-way ANOVA. *p < 0.05, ^#^*p* < 0.0001. Error bars are mean ± standard error of the mean (SEM).

Previous studies in mice have determined that contextual fear generalization at proximal time intervals (24–48 h after fear conditioning) is sensitive to both sex and the test order of the training and generalization contexts (Huckleberry et al., 2016; Keiser et al., 2017). Thus, we first examined the influence of sex and test order on the generalization of contextual fear at remote time-points (3 weeks after fear conditioning). In our initial experiments, we designed the training and generalization contexts to be as perceptually distinct as possible from one another to establish baseline levels of generalization (Figure 1). Male and female mice were conditioned in the training context (Context A) and then tested 21 days later to measure freezing in either Context A or the generalization context (Context B; Figure 2A). Both test orders (A→B and B→A) were evaluated in separate cohorts of mice, with a 24 h period between retrieval tests (Figure 2A).

We detected main effects of Test Context and Test Order on freezing behavior by three-way ANOVA, as well as a Test Context × Test Order interaction and Sex × Test Order interaction (Figure 2B; Test Context: *F*_(1,112)_ = 74.97, *p* < 0.0001; Test Order: *F*_(1,112)_ = 27.61, *p* < 0.0001; Test Context × Test Order: *F*_(1,112)_ = 30.16, *p* < 0.0001; Sex × Test Order: *F*_(1,112)_ = 20.10, *p* < 0.0001). Bonferroni *post hoc* comparisons indicated that male mice exhibited comparatively little freezing in the generalization context (Context B) vs. the training context (Context A) regardless of test order (Figure 2B; Males A→B: *p* < 0.0001 for freezing in Context A vs. Context B; Males B→A: *p* = 0.0485 for freezing in Context A vs. Context B), in agreement with earlier work examining contextual discrimination at 24–48 h (Keiser et al., 2017; but see Huckleberry et al., 2016). Female mice that were tested in Context A prior to Context B also exhibited relatively higher freezing in the training context, while those tested in the reverse test order (B→A) showed similar levels of freezing to both contexts (Figure 2B; Females A→B: *p* < 0.0001 for freezing in Context A vs. Context B). A more pronounced effect of test order in females than males was also observed for proximal time-points by Keiser et al. (2017).

To probe these effects further, we calculated a discrimination index based on the ratio of time spent freezing in each of the test contexts. This analysis confirmed our initial findings showing high levels of context discrimination [calculated as % time freezing in Context A/(% time freezing in Context A + Context B)] in the first three experimental groups (i.e., males A→B and B→A, and females A→B), while the female (B→A) cohort showed minimal departure from chance-level freezing. Analysis of the discrimination data by two-way ANOVA identified main effects of both Sex and Test Order, as well as a sex × test order interaction (Figure 2C; Sex: *F*_(1,56)_ = 6.45, *p* = 0.0139; Test Order: *F*_(1,56)_ = 15.35, *p* = 0.0002; Sex × Test Order: *F*_(1,56)_ = 16.06, *p* = 0.0002). Bonferroni *post hoc* comparisons revealed significant differences between the female (A→B) vs. (B→A) groups (*p* < 0.0001). Thus, in experimental paradigms employing completely distinct training and generalization contexts, only female mice tested in the (B→A) order were incapable of discriminating between contexts.

Finally, because there appeared to be additional meaningful comparisons in Figure 2B that were not detected by the original three-way ANOVA—in particular, freezing to Context B across experimental groups, and therefore generalization—we re-analyzed the effects of test order in males and females separately by two-way ANOVA to increase statistical power. For female mice, we again detected a main effect of Test Context as well as a Test Context × Test Order interaction by two-way ANOVA (Figure 2B; Test Context: *F*_(1,56)_ = 27.68, *p* < 0.0001; Test Context × Test Order: *F*_(1,56)_ = 18.85, *p* < 0.0001). Furthermore, Bonferroni *post hoc* comparisons revealed significant effects of test order on freezing in both Context A (*p* < 0.01) and Context B (*p* < 0.05). Analysis of male mice by two-way ANOVA identified main effects of Test Context and Test Order, as well as a Test Context × Test Order interaction (Figure 2B; Test Context: *F*_(1,56)_ = 47.83, *p* < 0.0001; Test Order: *F*_(1,56)_ = 43.09, *p* < 0.0001; Test Context × Test Order: *F*_(1,56)_ = 12.10, *p* = 0.0010). While the effect of Test Order on freezing in Context A was determined to be significant by Bonferroni’s *post hoc* test (*p* < 0.0001), freezing in Context B did not reach statistical significance (*p* = 0.0666). Thus, generalized freezing in Context B was more sensitive to test order for the female (B→A) group. In other words, within the parameters of this behavioral paradigm, female mice are predisposed to heightened freezing in a novel context that is presented before re-exposure to the training context, while males do not exhibit such a bias.

### Experiment 2: Remote Fear Generalization in Female Mice Requires Associative Contextual Fear Memory

CFC and fear generalization are associative processes, whereby an animal requires a minimum time of exposure to a context in order to form a unitary representation from stimulus elements and subsequently associate that unitary representation with a foot shock (Fanselow, 1986, 1990; Rudy et al., 2004; Rudy, 2009; Sauerhofer et al., 2012; Maren et al., 2013). Given the female-specific influence of test order on discrimination between distinct contexts in Experiment 1, we compared the impact of brief training vs. standard CFC on remote memory and generalization to determine if the effects of test order are dependent on the formation of an associative contextual fear memory.

**Figure 3:**
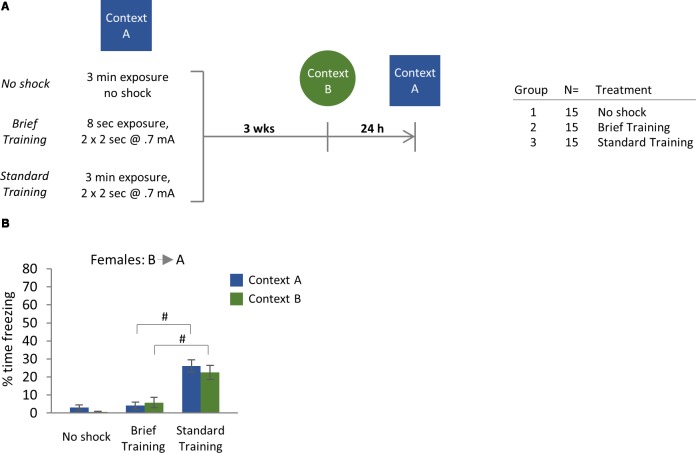
**(A)** Experimental design and cohort information. **(B)** Effect of fear conditioning protocol (cage-exposed mice with no shock vs. brief training protocol vs. standard CFC) in female mice (B→A test order) at 3 weeks after initial training. Significant Tukey *post hoc* comparisons following two-way ANOVA are indicated. ^#^*p* < 0.0001. Error bars are mean ± SEM.

Mice were trained in either the standard CFC protocol or exposed to brief training (Figure 3A), which normally does not produce associative memory (Fanselow, 1990). A third group of mice was exposed to Context A for 3 min in the absence of foot shocks. Analysis of the effect of conditioning protocol on freezing to Context A or Context B by two-way ANOVA indicated a main effect of Training Protocol (Figure 3B; Training Protocol: *F*_(2,84)_ = 43.25, *p* < 0.0001). In particular, with the brief training protocol, we observed minimal levels of freezing to either context. Moreover, the levels of freezing produced by the brief training protocol were not statistically different from those observed in control mice that were exposed to the fear conditioning chambers in the absence of shock. However, Tukey’s *post hoc* test revealed that freezing behavior produced by standard training was significantly greater than that produced by the brief training protocol (Figure 3B; *p* < 0.0001 for brief vs. standard training for either Context A or Context B). We conclude that, like contextual fear memory, the generalization of contextual fear at remote time points is an associative process for female mice in our paradigm.

### Experiment 3: Female (B→A) Mice Exhibit Context Discrimination at Proximal Intervals

**Figure 4:**
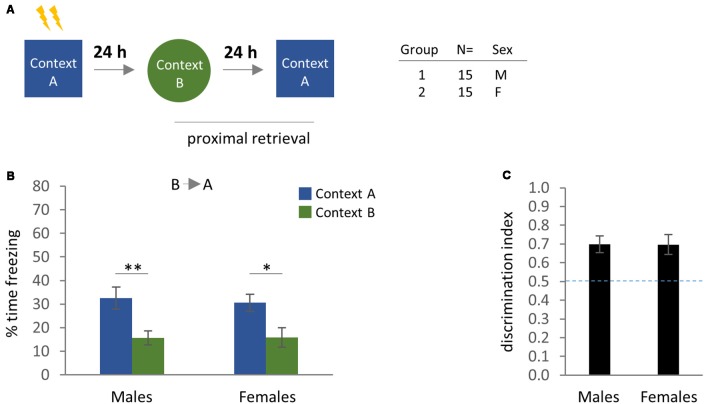
Contextual fear memory and generalization at proximal time-points (BA test order). **(A)** Experimental design and cohort information. **(B)** Comparison of freezing behavior in male and female mice at 24–48 h after conditioning, using distinct training and generalization contexts. Bonferroni *post hoc* comparisons following two-way ANOVA are indicated. **(C)** Discrimination index for males and females at 24–48 h after conditioning. *p < 0.05, **p < 0.01. Error bars are mean ± SEM.

In Experiment 1, we observed that female mice tested in Context B prior to Context A exhibited similar levels of freezing in both contexts, indicating poor context discrimination (Figures 2B,C). To determine whether the emergence of this phenotype was time-dependent, we performed the same experiment in female and male mice at proximal time intervals (Figure 4A). Here, a two-way ANOVA showed a main effect of Test Context (Figure 4B; Test Context: *F*_(1,56)_ = 16.32, *p* = 0.0002), but no significant effect of Sex. Bonferroni’s *post hoc* test determined that freezing in Context A vs. Context B was significant for both males (*p* = 0.0068) and females (*p* = 0.0208). Additionally, an unpaired *t-test* did not identify a significant difference in the discrimination index between males and females (Figure 4C). Therefore, although test order was an important factor in females at remote time intervals, female mice were perfectly capable of discriminating between training and generalization contexts at proximal time-points, consistent with the idea that the generalization of fear increases over time (Wiltgen and Silva, 2007).

### Experiment 4: Pre-exposure to the Training Context Enhances Context Discrimination in Females

**Figure 5:**
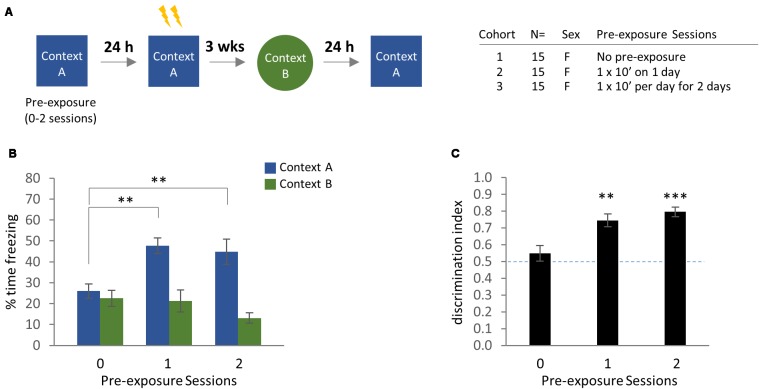
Effect of context pre-exposure (0–2 sessions) on remote contextual fear generalization using distinct training and generalization contexts. **(A)** Experimental design. *N* = 30 mice per test order (AB or BA), with 15 mice for each pre-exposure condition. **(B)** Effect of test order on freezing behavior in the training context vs. novel context. Significant Bonferroni *post hoc* comparisons following two-way ANOVA are indicated. **(C)** Discrimination index calculations from previous data. Significant Bonferroni *post hoc* comparisons following one-way ANOVA are shown in graph. Dotted line indicates no-discrimination threshold. **p < 0.01, ***p < 0.001. Error bars are mean ± SEM.

Given that remote fear generalization in females is dependent on the formation of an associative memory, we hypothesized that pre-exposure to the training context (Context A) may enhance context discrimination by improving contextual learning and strengthening the representation of Context A. In theory, pre-exposure should ameliorate the effects of test order and reduce the generalized freezing in Context B that we observed in female mice if, in fact, generalization resulted from forming a weaker representation of Context A (Fanselow, 1990; Urcelay and Miller, 2014). To examine these possibilities, female mice were pre-exposed to Context A for either a single 10 min session or two 10 min sessions on consecutive days prior to conditioning (Figure 5A). Control mice were not pre-exposed to Context A, but were fear conditioned as usual in Context A, followed by the same type and order of retrieval tests as the other experimental groups. Like previous experiments, freezing in Context A and Context B was evaluated 3 weeks later.

In comparing the effects of pre-exposure to the female (B→A) data from Experiment 1, we detected a significant main effect of Test Context by two-way ANOVA, as well as a trend for a main effect of Pre-exposure Sessions (Figure 5B; Test Context: *F*_(1,84)_ = 34.250, *p* < 0.0001; Pre-exposure Sessions: *F*_(2,84)_ = 2.837, *p* = 0.0642). In addition, we observed a significant interaction between Test Context and Pre-exposure Sessions (Figure 5B; Test Context × Pre-exposure Sessions: *F*_(2,84)_ = 6.106, *p* = 0.0033). Bonferroni *post hoc* correction demonstrated that pre-exposure caused a significant increase in freezing to Context A vs. no pre-exposure, although there was no difference between one or two pre-exposure sessions (Figure 5B; Context A: 1 pre-exposure vs. no pre-exposure, *p* = 0.0017; two pre-exposure sessions vs. no pre-exposure, *p* = 0.0076). We observed a modest, dose-dependent reduction in freezing to Context B as a function of pre-exposure sessions, but the effect did not survive *post hoc* testing.

In comparing discrimination indices as a function of Pre-exposure using a one-way ANOVA, we found a significant main effect of Pre-exposure sessions (Figure 5C; Pre-exposure Sessions: *F*_(2,42)_ = 11.370, *p* = 0.0001). Tukey’s multiple comparisons test revealed a significant increase in discrimination index with Pre-exposure vs. without Pre-exposure (Figure 5C; No Pre-exposure vs. 1 Pre-exposure Session, *p* = 0.0025; no Pre-exposure vs. 2 Pre-exposure Sessions: *p* = 0.0001), but there was no difference between 1 or 2 Pre-exposure Sessions. Therefore, the ability of female mice to discriminate between contexts presented in the (B→A) test order was greatly enhanced by pre-exposure, and this effect was predominantly driven by improved contextual fear memory for the training context, rather than by a reduction in generalized freezing to Context B. These results suggest that pre-exposure enables female mice to form a more detailed contextual representation of the training context, which in turn supports greater memory precision.

### Experiment 5: Tactile Contextual Elements Promote Generalization of Remote Contextual Fear and Reduce Discrimination in Males

**Figure 6:**
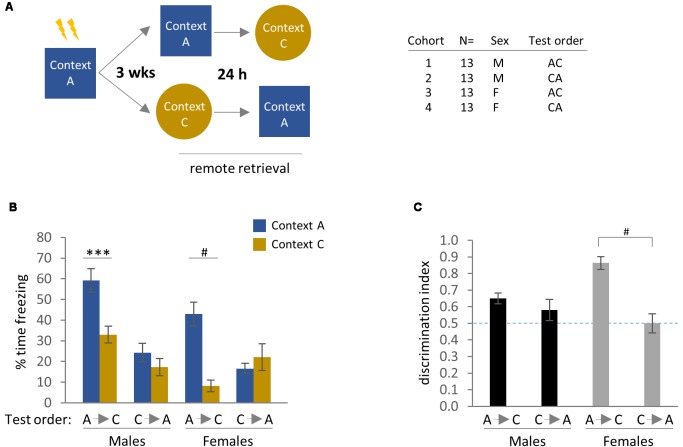
Remote contextual fear generalization using context that retains metal grid floor used in the training context. **(A)** Experimental design. Context A and Context C are completely different in terms of odor, chamber shape, and lighting. However, the same metal grid floor through which shocks were delivered during training is present in both contexts. **(B)** Freezing behavior of male and female mice in remote contextual generalization, with both test orders (A→C and C→A). Significant Bonferroni *post hoc* effects following three-way ANOVA are indicated. **(C)** Discrimination index calculated from freezing data, with Bonferroni *post hoc* test following two-way ANOVA revealed a significant effect of test order in females. ***p < 0.001 and ^#^*p* < 0.0001. Error bars are mean ± SEM.

As indicated earlier, the contexts used in the previous experiments were designed to be as distinct as possible to establish baseline levels of contextual fear generalization. We next asked if manipulating particular features between the training and generalization contexts could influence generalization aside from test order. A particularly salient feature in CFC is tactile information provided by the grid floor through which foot shocks are delivered (Huckleberry et al., 2016). Therefore, we examined whether inclusion of this contextual element in a novel test context C that was otherwise completely different from the training context would have an impact on a remote generalization (Figure 6A).

In this experiment, we detected main effects of Test Context, Test Order, and Sex by three-way ANOVA, as well as several two-way interactions between these factors (Figure 6B; Test Context: *F*_(1,96)_ = 22.40, *p* < 0.0001; Test Order: *F*_(1,96)_ = 22.96, *p* < 0.0001; Sex: *F*_(1,96)_ = 11.13, *p* = 0.0012; Test Context × Test Order: *F*_(1,96)_ = 20.25, *p* < 0.0001; Test Order × Sex: *F*_(1,96)_ = 8.24, *p* = 0.0050). A three-way interaction fell short of statistical significance (*p* = 0.1153), while Bonferroni *post hoc* comparisons identified significant effects of Test Context for males and females in the (A→C) groups (Figure 6B; Males: *p* = 0.0006; Females: *p* < 0.0001). Analysis of discrimination indices by two-way ANOVA revealed a main effect of Test Order as well as an interaction between Test Order and Sex (Figure 6C; Test Order: *F*_(1,48)_ = 20.08, *p* < 0.0001; Test Order × Sex: *F*_(1,96)_ = 9.28, *p* = 0.0038). In addition, Bonferroni *post hoc* comparisons identified a significant effect of Test Order for females only (*p* < 0.0001).

As in Experiment 1, we also examined the effects of test order on freezing to Context A and Context C within male and female groups using a two-way ANOVA to increase statistical power. Analysis of female mice revealed a main effect of Test Context as well as a Test Order × Test Context interaction (Figure 6B; Test Context: *F*_(1,48)_ = 9.624, *p* = 0.0032; Test Order × Test Context: *F*_(1,48)_ = 18.13, *p* < 0.0001), while Bonferroni’s *post hoc* comparison indicated a significant effect of test order on freezing to Context A (*p* = 0.0005) and a trend for freezing to Context C (*p* = 0.0895). For males, two-way ANOVA detected main effects of Test Context and Test Order, as well as an interaction between these parameters (Figure 6B; Test Context: *F*_(1,48)_ = 12.94, *p* = 0.0008; Test Order:* F*_(1,48)_ = 30.02, *p* < 0.0001; Test Order × Test Context: *F*_(1,48)_ = 4.33, *p* = 0.0427). Bonferroni’s *post hoc* test recovered significant effects of test order on freezing in both Context A (*p* < 0.0001) and Context C (*p* = 0.0404).

In summary, both male and female mice were able to discriminate between Context A and Context C when presented in the (A→C) test order, although the overall discrimination index for males was markedly lower than in the previous experiments utilizing completely distinct contexts (compare Figures 2C, 6C). However, for both males and females, context discrimination was abolished by testing in the reverse order. This observation is consistent with earlier work demonstrating the importance of tactile elements in driving context discrimination at proximal time intervals in male mice (Huckleberry et al., 2016). On the other hand, female mice in the (A→C) group showed robust context discrimination, suggesting that tactile features are much less salient for females than for males at remote time intervals. Furthermore, test order became a significant variable for males only when the generalization context retained salient features of the training context (Figure 6), but not when contexts were sufficiently distinct (Figure 2).

### Experiment 6: Tactile Contextual Elements Promote Generalization of Proximal Contextual Fear and Reduce Discrimination in Males and Females in the (C→A) Test Order

**Figure 7:**
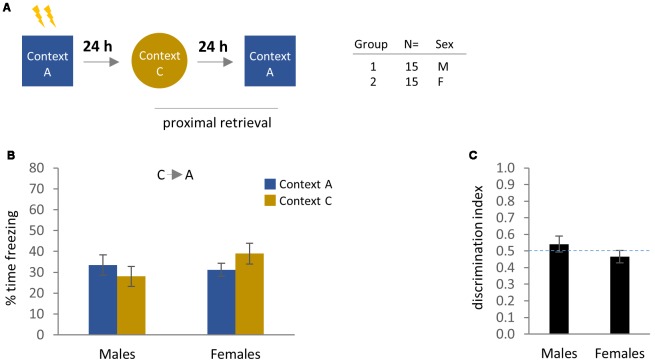
Contextual fear memory and generalization at proximal time-points (C→A test order), in which the metal grid floor of the training context is retained in the otherwise contextually distinct generalization context. **(A)** Experimental design and cohort information. **(B)** Comparison of freezing behavior in male and female mice at 24–48 h after conditioning. **(C)** Discrimination index. Error bars are mean ± SEM.

Given the generalized fear and absence of contextual discrimination observed in both males and females in the (C→A) group in Figure 6, we next evaluated whether such behavioral patterns are likewise present in the 24–48 h (Figure 7A) following initial CFC, or whether they develop over time. We observed that both males and females exhibited similar levels of freezing in Context A and Context C (Figure 7B), with no evidence of contextual discrimination (Figure 7C). Therefore, tactile information provided by the metal grid floor in an otherwise distinct context (Context C) is sufficient to promote levels of freezing similar to what we observed for the training context (Context A), at least for the (C→A) test order.

## Discussion

Contextual fear generalization and context discrimination at remote time-points are strongly influenced by several factors, including the saliency of specific contextual features, test order, and sex differences. Furthermore, these experimental variables interact to modulate behavior. The key findings of our studies over remote time intervals are as follows: (1) female mice are predisposed to exhibiting generalized fear in the first context that they encounter at remote time points after CFC, as well as poor context discrimination, even if the training and testing contexts are perceptually distinct; (2) the latter effects require the formation of an associative memory, and emerge over time; (3) for female mice, pre-exposure improves discrimination primarily by enhancing memory for the training context, rather than by reducing generalization; (4) both male and female mice exhibit greater freezing in the training context when presented before vs. after the generalization context, which may involve reconsolidation and interference rather than inter-trial extinction; and (5) tactile cues are more salient for male mice than for females.

### Test Order Influences Remote Fear Generalization in Females

In our experiments, female mice exhibited generalized fear at remote time-points when first tested in a non-reinforced generalization context (Context B or C). This finding builds on previous observations showing an effect of test order at proximal time-points (Huckleberry et al., 2016; Keiser et al., 2017). However, in contrast to the latter studies, our observations were made using a generalization context (Context B) that was designed to be as perceptually distinct as possible from the training context (Context A). In fact, female mice showed robust differences in freezing in the training and generalization contexts as a function of test order, irrespective of whether the generalization context was distinct from, or shared at least one important contextual feature with, the training context. In addition, these effects were particularly apparent when evaluated in terms of discrimination indices, which permitted control over inter-individual variability in freezing levels. For male mice, despite the fact that overall differences in freezing levels varied as a function of a test order, remote discrimination between Context A and Context B was unaffected by test order and remained high. We conclude that male mice are, overall, less sensitive than females to the effects of a test order.

What can explain these observations? It is unlikely that the effect of test order is produced by sex differences in US processing because manual scoring of shock responsivity (e.g., running and jumping behavior) revealed no significant differences between males and females (data not shown). In addition, differences in inter-trial extinction are unlikely to be a contributing factor, because a single test session lasting only 3 min is not sufficient to support extinction (Lattal and Maughan, 2012), while the large difference in freezing levels in the training vs. generalization context in the (A→B or C) test order would entail a far greater rate of contextual fear extinction than one would typically observe in mice. Furthermore, the potential role of extinction is rendered all the more improbable by the substantial perceptual differences of the training and generalization contexts.

It is also unlikely that cues present during transport or from the experimenter strongly influenced our findings because female mice exhibited low levels of freezing in the generalization context in the (A→B or C) test order, even though both the experimenter and transport cues remained static. Moreover, in the brief training protocol, which emphasizes the salience of extra-contextual cues and features relative to the conditioning context, female mice did not show significant freezing to the generalization context. While we cannot fully rule out the possibility that transport cues or the experimenter did not in some way act as “occasion setters” for heightened conditioned freezing exhibited by female mice upon placement in the first testing context (Holland, 1992), we would not expect to observe such low levels of freezing in the generalization context if extra-contextual information were important.

Although animals were not trained to asymptotic levels of freezing, another potential explanation for the bi-directional shift in freezing in the B→A or C test order is that the reinforced training context shared associative strength with the non-reinforced generalization context. However, this interpretation is also unlikely given that the environments (i.e., Context B vis-à-vis Context A) were as different as possible along tactile, olfactory, visual, and spatial dimensions. While earlier work demonstrates a time-dependent increase in generalization irrespective of test order and without a reduction in freezing to the training context (Wiltgen and Silva, 2007), it is important to recognize that differences in procedural variables such as fear conditioning parameters (e.g., shock number, intensity, and delivery schedule) and test design (e.g., similarity between training and generalization contexts, and timing of retrieval tests), as well as mouse genetic background, may preclude rigorous comparison of studies.

In our study, we speculate that the effect of test order in females may result from inadequate CS learning (see Spence, 1936) coupled with a mismatch between the expected and actual outcome, as captured by Pearce-Hall, Rescorla-Wagner, and Temporal-Difference theoretical models (Rescorla and Wagner, 1972; Pearce and Hall, 1980; Sutton, 1988). This hypothesis is supported by the fact that (1) our effects depended on associative learning; and (2) increasing pre-exposure to the training context, and thus increasing learning about the CS, ameliorated the test order effect, primarily by enhancing the strength of the context-specific fear memory. The reduced levels of freezing shown by females vs. males in Context A when tested prior to the generalization context are consistent with the idea of a weaker contextual representation. Although female mice can form a contextual representation of the training context, such a representation becomes more detailed as a consequence of pre-exposure (Rudy and O’Reilly, 1999; Keiser et al., 2017), which may be driven by learning-related structural changes in key hippocampal circuits that support memory precision (Ruediger et al., 2011). Finally, individuals with PTSD show greater reactivity to prediction errors, while females, in particular, show a greater difficulty with the encoding of prediction errors (Ross et al., 2018; Homan et al., 2019). Thus, it is plausible that fear generalization in female mice is a product of inadequate CS learning compounded by changes in prediction error.

### Tactile Features Are More Salient for Males

Previous work has shown that tactile and olfactory elements exert the most powerful influence over the generalization of contextual fear at proximal time-points in males (Huckleberry et al., 2016). Thus, we also explored the consequences of retaining the metal grid floor in the generalization context (Context C) at a remote time-point. While this manipulation strongly inhibited remote contextual discrimination in male mice, females continued to exhibit strong discrimination between the training and generalization contexts as long as the training context was presented first. In other words, whereas female mice only exhibited heightened freezing in the generalization context when tested first—regardless of perceptual features—males showed pronounced generalization only when the training and generalization contexts shared at least one perceptual element (i.e., tactile cues provided by the metal grid). Thus, our observations support the notion that tactile features are, overall, more salient for males than for females. Finally, both males and females failed to exhibit context discrimination at proximal intervals when the generalization context (Context C) was presented first. Therefore, the absence of context discrimination at remote intervals with the latter test order is not a phenomenon that emerges over time, in contrast to what we observed in behavioral experiments using distinct training and generalization contexts.

### Role of Interference and Reconsolidation on Test Order Effects

Both male and female mice exhibited heightened freezing to the training context at remote time intervals when tested prior to the generalization context, in comparison to the reverse order. Furthermore, we observed this effect for both generalization contexts (Contexts B and C). For reasons stated earlier, we would argue against inter-trial extinction as a contributing factor in the observed behavioral outputs. Instead, fear generalization is modulated by proactive and retroactive interference produced by exposure to novel contexts (Besnard and Sahay, 2016), and it is possible that initial exposure to the non-reinforced generalization context could drive a reassignment in cue value that produces a concomitant reduction of freezing to the training context. Initial exposure to the generalization context may support partial reactivation of the aversive training memory that is subsequently reconsolidated in an attenuated form. Conversely, initial testing in the training context serves as a reminder that promotes memory accuracy and therefore improves discrimination when animals are subsequently exposed to the generalization context (De Oliveira Alvares et al., 2013). However, the neurobiological mechanisms governing reconsolidation at remote time intervals are likely to be distinct from those operating at proximal time-points, when context discrimination remains high for female mice. Post-discrimination shifts in generalization gradients represent another potential mechanism by which freezing in the training context at remote time-points may be reduced by prior testing in the generalization context (ten Cate and Rowe, 2007). Furthermore, we speculate that memory storage processes such as pattern completion and reconsolidation may be differentially engaged among males and females (Rolls, 2013), as well as stress-induced alterations (Zoladz et al., 2011) on systems-level processes that operate during remote memory retrieval (Asok et al., 2019b). Additional experiments are needed to investigate these and other explanations.

### Evolutionary Implications of Test Order Effects

The proclivity to exhibit a heightened fear response in the first context presented after an aversive event may represent an optimal evolutionary strategy for female mice (Kelley, 1988; Huckleberry et al., 2016; Bangasser and Wicks, 2017). For example, although an inappropriate or excessive defensive response may interfere with the acquisition of resources obtained through potentially risky behaviors such as foraging, such a strategy of erring on the side of safety is more likely to ensure reproductive success in the long run. In this regard, the increased generalization of contextual fear observed in female mice at remote time-points supports the idea that the selection of an optimal defensive strategy is sex-dependent (Gruene et al., 2015; Shansky, 2018). In the absence of sex differences in US processing, our findings are consistent with the idea that the CS or context representation is weaker in females. Importantly, in our study, conditioning parameters such as context placement-to-shock interval as well as the shock intensity and shock duration, produced levels of freezing comparable to studies that use a single-trial conditioning paradigm (Fanselow, 1986, 1990; Wiltgen et al., 2001). Finally, sex differences in generalization and context discrimination at proximal intervals are thought to reflect a differential recruitment of hippocampal and amygdalar circuitry (Keiser et al., 2017). This is likely true for remote memories and warrants further investigation.

### PTSD

Animal models based on fear conditioning have generated a wealth of elementary knowledge into the molecular and neural circuit mechanisms that mediate the storage and retrieval of aversive memory (Schafe et al., 2001; Maren et al., 2013). Such studies have provided a useful framework with which to understand how the aberrant processing of fear memory may contribute to psychopathological changes observed in PTSD (Ross et al., 2017; Norrholm and Jovanovic, 2018; Zuj and Norrholm, 2019). Given that PTSD is a disorder of fear memory (McNally, 2006; Ross et al., 2017), and that fear is highly conserved throughout the animal kingdom (LeDoux, 2012; Adolphs, 2013), it is plausible that fear conditioning-based studies in rodents can reveal causative pathological mechanisms that govern the development of PTSD. However, we acknowledge that fear conditioning *per se* does not represent a complete model of PTSD. At best, animal studies can only model sub-components (i.e., intermediate phenotypes and endophenotypes) of these disorders, some of which are nonetheless highly amenable to experimentation in animals, such as fear generalization. Indeed, the generalization of contextual fear in humans is a relatively underexplored area of research (Andreatta et al., 2015), and our studies in mice should inform the design of experiments with human subjects.

### Summary

Our findings reveal behavioral and parametric constraints of fear generalization and context discrimination in female mice at remote time-points. These findings also highlight how sex differences in the acquisition, consolidation, or retrieval of contextual representations may influence defensive behaviors to neutral environments long after the learning has occurred. Moreover, our findings point to the need for a better understanding of how contextual processing differs between sexes in hippocampal subfields to promote or prevent remote fear generalization, and how such differences might contribute to overgeneralization. Alterations in contextual processing have been proposed as a core feature in disorders including PTSD (Maren et al., 2013). Future studies that examine how hippocampal circuits interact with cortical networks to encode, store, and maintain contextual representations during systems consolidation will provide significant insights into the sex-specific neurobiological mechanisms of psychopathological disorders such as PTSD.

## Data Availability

All datasets generated for this study are included in the manuscript.

## Author Contributions

JR and AA conceived and wrote the manuscript. JH and LH performed the experiments. JR, AA and JH analyzed the data. SK and EK helped prepare the manuscript and provided key conceptual insights.

## Conflict of Interest Statement

The authors declare that the research was conducted in the absence of any commercial or financial relationships that could be construed as a potential conflict of interest.

## References

[B1] Adolphs R. (2013). The biology of fear. Curr. Biol. 23, R79–R93. 10.1016/j.cub.2012.11.055 23347946 PMC3595162

[B2] Andreatta M. Leombruni E. Glotzbach-Schoon E. Pauli P. Muhlberger A. (2015). Generalization of contextual fear in humans. Behav. Ther. 46, 583–596. 10.1016/j.beth.2014.12.008 26459839

[B3] APA (2013). Diagnostic and Statistical Manual of Mental Disorders, Fifth Edition. Arlington, VA: American Psychiatric Association.

[B4] Asok A. Kandel E. R. Rayman J. B. (2019a). The neurobiology of fear generalization. Front. Behav. Neurosci. 12:329. 10.3389/fnbeh.2018.00329 30697153 PMC6340999

[B5] Asok A. Leroy F. Rayman J. B. Kandel E. R. (2019b). Molecular mechanisms of the memory trace. Trends Neurosci. 42, 14–22. 10.1016/j.tins.2018.10.005 30391015 PMC6312491

[B6] Bangasser D. A. Wicks B. (2017). Sex-specific mechanisms for responding to stress. J. Neurosci. Res. 95, 75–82. 10.1002/jnr.23812 27870416 PMC5120612

[B7] Besnard A. Sahay A. (2016). Adult hippocampal neurogenesis, fear generalization, and stress. Neuropsychopharmacology 41, 24–44. 10.1038/npp.2015.167 26068726 PMC4677119

[B8] Biedenkapp J. C. Rudy J. W. (2007). Context preexposure prevents forgetting of a contextual fear memory: implication for regional changes in brain activation patterns associated with recent and remote memory tests. Learn. Mem. 14, 200–203. 10.1101/lm.499407 17351145 PMC2519802

[B9] Blanchard R. J. Blanchard C. D. (1989). Attack and defense in rodents as ethoexperimental models for the study of emotion. Prog. Neuropsychopharmacol. Biol. Psychiatry 13, S3–S14. 10.1016/0278-5846(89)90105-x 2694228

[B10] Blume S. R. Freedberg M. Vantrease J. E. Chan R. Padival M. Record M. J.. (2017). Sex- and estrus-dependent differences in rat basolateral amygdala. J. Neurosci. 37, 10567–10586. 10.1523/JNEUROSCI.0758-17.2017 28954870 PMC5666581

[B11] Byers S. L. Wiles M. V. Dunn S. L. Taft R. A. (2012). Mouse estrous cycle identification tool and images. PLoS One 7:e35538. 10.1371/journal.pone.0035538 22514749 PMC3325956

[B12] Day H. L. L. Reed M. M. Stevenson C. W. (2016). Sex differences in discriminating between cues predicting threat and safety. Neurobiol. Learn. Mem. 133, 196–203. 10.1016/j.nlm.2016.07.014 27423522 PMC4993817

[B13] De Oliveira Alvares L. Crestani A. P. Cassini L. F. Haubrich J. Santana F. Quillfeldt J. A. (2013). Reactivation enables memory updating, precision-keeping and strengthening: exploring the possible biological roles of reconsolidation. Neuroscience 244, 42–48. 10.1016/j.neuroscience.2013.04.005 23587841

[B14] Dudai Y. Karni A. Born J. (2015). The consolidation and transformation of memory. Neuron 88, 20–32. 10.1016/j.neuron.2015.09.004 26447570

[B15] Dunsmoor J. E. Paz R. (2015). Fear generalization and anxiety: behavioral and neural mechanisms. Biol. Psychiatry 78, 336–343. 10.1016/j.biopsych.2015.04.010 25981173

[B16] Dymond S. Dunsmoor J. E. Vervliet B. Roche B. Hermans D. (2015). Fear generalization in humans: systematic review and implications for anxiety disorder research. Behav. Ther. 46, 561–582. 10.1016/j.beth.2014.10.001 26459838

[B17] Fanselow M. S. (1986). Associative vs. topographical accounts of the immediate shock-freezing deficit in rats: implications for the response selection rules governing species-specific defensive reactions. Learn. Motiv. 17, 16–39. 10.1016/0023-9690(86)90018-4

[B18] Fanselow M. S. (1990). Factors governing one-trial contextual conditioning. Anim. Learn. Behav. 18, 264–270. 10.3758/bf03205285

[B19] Fanselow M. S. Lester L. S. (1988). “A functional behavioristic approach to aversively motivated behavior: predatory imminence as a determinant of the topography of defensive behavior,” in Evolution and Learning, eds Bolles R. C. Beecher M. D. (Hillsdale, NJ: Lawrence Erlbaum Associates, Inc.), 185–211.

[B20] Grillon C. Pine D. S. Lissek S. Rabin S. Bonne O. Vythilingam M. (2009). Increased anxiety during anticipation of unpredictable aversive stimuli in posttraumatic stress disorder but not in generalized anxiety disorder. Biol. Psychiatry 66, 47–53. 10.1016/j.biopsych.2008.12.028 19217076 PMC2696581

[B21] Gruene T. M. Flick K. Stefano A. Shea S. D. Shansky R. M. (2015). Sexually divergent expression of active and passive conditioned fear responses in rats. Elife 4:e11352. 10.7554/elife.11352 26568307 PMC4709260

[B22] Holland P. C. (1992). “Occasion setting in Pavlovian conditioning,” in Psychology of Learning and Motivation, eds Schmajuk N. A. Holland P. C. (San Diego, CA: Academic Press), 69–125.

[B23] Homan P. Levy I. Feltham E. Gordon C. Hu J. Li J.. (2019). Neural computations of threat in the aftermath of combat trauma. Nat. Neurosci. 22, 470–476. 10.1038/s41593-018-0315-x 30664770 PMC6829910

[B24] Huckleberry K. A. Ferguson L. B. Drew M. R. (2016). Behavioral mechanisms of context fear generalization in mice. Learn. Mem. 23, 703–709. 10.1101/lm.042374.116 27918275 PMC5110986

[B25] Jasnow A. M. Lynch J. F. III. Gilman T. L. Riccio D. C. (2017). Perspectives on fear generalization and its implications for emotional disorders. J. Neurosci. Res. 95, 821–835. 10.1002/jnr.23837 27448175

[B26] Jovanovic T. Kazama A. Bachevalier J. Davis M. (2012). Impaired safety signal learning may be a biomarker of PTSD. Neuropharmacology 62, 695–704. 10.1016/j.neuropharm.2011.02.023 21377482 PMC3146576

[B27] Jovanovic T. Norrholm S. D. Blanding N. Q. Davis M. Duncan E. Bradley B.. (2010). Impaired fear inhibition is a biomarker of PTSD but not depression. Depress. Anxiety 27, 244–251. 10.1002/da.20663 20143428 PMC2841213

[B28] Kaczkurkin A. N. Burton P. C. Chazin S. M. Manbeck A. B. Espensen-Sturges T. Cooper S. E.. (2017). Neural substrates of overgeneralized conditioned fear in PTSD. Am. J. Psychiatry 174, 125–134. 10.1176/appi.ajp.2016.15121549 27794690 PMC7269602

[B29] Keiser A. A. Turnbull L. M. Darian M. A. Feldman D. E. Song I. Tronson N. C. (2017). Sex differences in context fear generalization and recruitment of hippocampus and amygdala during retrieval. Neuropsychopharmacology 42, 397–407. 10.1038/npp.2016.174 27577601 PMC5399239

[B30] Kelley D. B. (1988). Sexually dimorphic behaviors. Annu. Rev. Neurosci. 11, 225–251. 10.1146/annurev.neuro.11.1.225 3284441

[B31] Kessler R. C. Chiu W. T. Demler O. Merikangas K. R. Walters E. E. (2005). Prevalence, severity, and comorbidity of 12-month DSM-IV disorders in the national comorbidity survey replication. Arch. Gen. Psychiatry 62, 617–627. 10.1001/archpsyc.62.6.617 15939839 PMC2847357

[B32] Lattal K. M. Maughan D. K. (2012). A parametric analysis of factors affecting acquisition and extinction of contextual fear in C57BL/6 and DBA/2 mice. Behav. Processes 90, 49–57. 10.1016/j.beproc.2012.03.008 22465469 PMC3337703

[B33] LeDoux J. (2012). Rethinking the emotional brain. Neuron 73, 653–676. 10.1016/j.neuron.2012.02.004 22365542 PMC3625946

[B34] Leer A. Sevenster D. Lommen M. J. J. (2018). Generalisation of threat expectancy increases with time. Cogn. Emot. [Epub ahead of print]. 10.1080/02699931.2018.1526167. 30260292

[B35] Liberzon I. Ressler K. (2016). Neurobiology of PTSD: From Brain to Mind. New York, NY: Oxford University Press.

[B37] Lissek S. Rabin S. Heller R. E. Lukenbaugh D. Geraci M. Pine D. S.. (2010). Overgeneralization of conditioned fear as a pathogenic marker of panic disorder. Am. J. Psychiatry 167, 47–55. 10.1176/appi.ajp.2009.09030410 19917595 PMC2806514

[B36] Lissek S. van Meurs B. (2015). Learning models of PTSD: theoretical accounts and psychobiological evidence. Int. J. Psychophysiol. 98, 594–605. 10.1016/j.ijpsycho.2014.11.006 25462219 PMC4809259

[B38] Lopresto D. Schipper P. Homberg J. R. (2016). Neural circuits and mechanisms involved in fear generalization: implications for the pathophysiology and treatment of posttraumatic stress disorder. Neurosci. Biobehav. Rev. 60, 31–42. 10.1016/j.neubiorev.2015.10.009 26519776

[B39] Lynch J. F. III. Cullen P. K. Jasnow A. M. Riccio D. C. (2013). Sex differences in the generalization of fear as a function of retention intervals. Learn. Mem. 20, 628–632. 10.1101/lm.032011.113 24131793

[B40] Lynch J. F. III. Winiecki P. Vanderhoof T. Riccio D. C. Jasnow A. M. (2016). Hippocampal cytosolic estrogen receptors regulate fear generalization in females. Neurobiol. Learn. Mem. 130, 83–92. 10.1016/j.nlm.2016.01.010 26851128

[B41] Maeng L. Y. Milad M. R. (2017). Post-traumatic stress disorder: the relationship between the fear response and chronic stress. Chronic Stress 1:2470547017713297 10.1177/2470547017713297 PMC7219872 32440579

[B42] Maren S. (2001). Neurobiology of Pavlovian fear conditioning. Annu. Rev. Neurosci. 24, 897–931. 10.1146/annurev.neuro.24.1.897 11520922

[B43] Maren S. Phan K. L. Liberzon I. (2013). The contextual brain: implications for fear conditioning, extinction and psychopathology. Nat. Rev. Neurosci. 14, 417–428. 10.1038/nrn3492 23635870 PMC5072129

[B44] McNally R. J. (2006). Cognitive abnormalities in post-traumatic stress disorder. Trends Cogn. Sci. 10, 271–277. 10.1016/j.tics.2006.04.007 16697695

[B45] Meziane H. Ouagazzal A. M. Aubert L. Wietrzych M. Krezel W. (2007). Estrous cycle effects on behavior of C57BL/6J and BALB/cByJ female mice: implications for phenotyping strategies. Genes Brain Behav. 6, 192–200. 10.1111/j.1601-183x.2006.00249.x 16827921

[B46] Milad M. R. Igoe S. A. Lebron-Milad K. Novales J. E. (2009). Estrous cycle phase and gonadal hormones influence conditioned fear extinction. Neuroscience 164, 887–895. 10.1016/j.neuroscience.2009.09.011 19761818 PMC2783784

[B47] Mobbs D. Hagan C. C. Dalgleish T. Silston B. Prévost C. J. (2015). The ecology of human fear: survival optimization and the nervous system. Front. Neurosci. 9:55. 10.3389/fnins.2015.00055 25852451 PMC4364301

[B48] Nemeroff C. B. Bremner J. D. Foa E. B. Mayberg H. S. North C. S. Stein M. B. (2006). Posttraumatic stress disorder: a state-of-the-science review. J. Psychiatr. Res. 40, 1–21. 10.1016/j.jpsychires.2005.07.005 16242154

[B49] Norrholm S. D. Jovanovic T. (2018). Fear processing, psychophysiology, and PTSD. Harv. Rev. Psychiatry 26, 129–141. 10.1097/hrp.0000000000000189 29734227

[B50] O’Reilly R. C. Rudy J. W. (2001). Conjunctive representations in learning and memory: principles of cortical and hippocampal function. Psychol. Rev. 108, 311–345. 10.1037//0033-295x.108.2.311 11381832

[B51] Parsons R. G. Ressler K. J. (2013). Implications of memory modulation for post-traumatic stress and fear disorders. Nat. Neurosci. 16, 146–153. 10.1038/nn.3296 23354388 PMC3752300

[B52] Pearce J. M. Hall G. (1980). A model for Pavlovian learning: variations in the effectiveness of conditioned but not of unconditioned stimuli. Psychol. Rev. 87, 532–553. 10.1037/0033-295x.87.6.532 7443916

[B53] Poulos A. M. Mehta N. Lu B. Amir D. Livingston B. Santarelli A.. (2016). Conditioning-and time-dependent increases in context fear and generalization. Learn. Mem. 23, 379–385. 10.1101/lm.041400.115 27317198 PMC4918784

[B54] Rescorla R. A. Wagner A. R. (1972). “A theory of Pavlovian conditioning: variations in the effectiveness of reinforcement and nonreinforcement,” in Classical Conditioning II: Current Research and Theory, (Vol. 2) eds Black A. H. Prokasy W. F. (New York, NY: Appleton-Century-Crofts), 64–99.

[B55] Rolls E. T. (2013). The mechanisms for pattern completion and pattern separation in the hippocampus. Front. Syst. Neurosci. 7:74. 10.3389/fnsys.2013.00074 24198767 PMC3812781

[B56] Ross D. A. Arbuckle M. R. Travis M. J. Dwyer J. B. van Schalkwyk G. I. Ressler K. J. (2017). An integrated neuroscience perspective on formulation and treatment planning for posttraumatic stress disorder: an educational review. JAMA Psychiatry 74, 407–415. 10.1001/jamapsychiatry.2016.3325 28273291 PMC5504531

[B57] Ross M. C. Lenow J. K. Kilts C. D. Cisler J. M. (2018). Altered neural encoding of prediction errors in assault-related posttraumatic stress disorder. J. Psychiatr. Res. 103, 83–90. 10.1016/j.jpsychires.2018.05.008 29783079 PMC6008230

[B59] Rudy J. W. (2009). Context representations, context functions, and the parahippocampal-hippocampal system. Learn. Mem. 16, 573–585. 10.1101/lm.1494409 19794181 PMC2769166

[B58] Rudy J. Huff N. Matus-Amat P. (2004). Understanding contextual fear conditioning: insights from a two-process model. Neurosci. Biobehav. Rev. 28, 675–685. 10.1016/j.neubiorev.2004.09.004 15555677

[B60] Rudy J. W. O’Reilly R. C. (1999). Contextual fear conditioning, conjunctive representations, pattern completion, and the hippocampus. Behav. Neurosci. 113, 867–880. 10.1037//0735-7044.113.5.867 10571471

[B61] Ruediger S. Vittori C. Bednarek E. Genoud C. Strata P. Sacchetti B.. (2011). Learning-related feedforward inhibitory connectivity growth required for memory precision. Nature 473, 514–518. 10.1038/nature09946 21532590

[B62] Sauerhofer E. Pamplona F. A. Bedenk B. Moll G. H. Dawirs R. R. von Hörsten S.. (2012). Generalization of contextual fear depends on associative rather than non-associative memory components. Behav. Brain Res. 233, 483–493. 10.1016/j.bbr.2012.05.016 22659395

[B63] Schafe G. E. Nader K. Blair H. T. LeDoux J. E. (2001). Memory consolidation of Pavlovian fear conditioning: a cellular and molecular perspective. Trends Neurosci. 24, 540–546. 10.1016/s0166-2236(00)01969-x 11506888

[B64] Shansky R. M. (2018). Sex differences in behavioral strategies: avoiding interpretational pitfalls. Curr. Opin. Neurobiol. 49, 95–98. 10.1016/j.conb.2018.01.007 29414071

[B65] Spence K. W. (1936). The nature of discrimination learning in animals. Psychol. Rev. 43, 427–449. 10.1037/h0056975 14912194

[B66] Starita F. Kroes M. Davachi L. Phelps E. Dunsmoor J. (2019). Threat learning promotes generalization of episodic memory. J. Exp. Psychol. Gen. [Epub ahead of print]. 10.1037/xge0000551 30667260 PMC6642861

[B67] Sutton R. S. (1988). Learning to predict by the methods of temporal differences. Mach. Learn. 3, 9–44. 10.1007/bf00115009

[B68] ten Cate C. Rowe C. (2007). Biases in signal evolution: learning makes a difference. Trends Ecol. Evol. 22, 380–387. 10.1016/j.tree.2007.03.006 17379354

[B69] Tolin D. F. Foa E. B. (2006). Sex differences in trauma and posttraumatic stress disorder: a quantitative review of 25 years of research. Psychol. Bull. 132, 959–992. 10.1037/0033-2909.132.6.959 17073529

[B70] Tronel S. Milekic M. H. Alberini C. M. (2005). Linking new information to a reactivated memory requires consolidation and not reconsolidation mechanisms. PLoS Biol. 3:e293. 10.1371/journal.pbio.0030293 16104829 PMC1188238

[B71] Urcelay G. P. Miller R. R. (2014). The functions of contexts in associative learning. Behav. Processes 104, 2–12. 10.1016/j.beproc.2014.02.008 24614400 PMC4011978

[B73] Wiltgen B. J. Sanders M. J. Behne N. S. Fanselow M. S. (2001). Sex differences, context preexposure and the immediate shock deficit in Pavlovian context conditioning with mice. Behav. Neurosci. 115, 26–32. 10.1037/0735-7044.115.1.26 11256449

[B72] Wiltgen B. J. Silva A. J. (2007). Memory for context becomes less specific with time. Learn. Mem. 14, 313–317. 10.1101/lm.430907 17522020

[B74] Wiltgen B. J. Zhou M. Cai Y. Balaji J. Karlsson M. G. Parivash S. N.. (2010). The hippocampus plays a selective role in the retrieval of detailed contextual memories. Curr. Biol. 20, 1336–1344. 10.1016/j.cub.2010.06.068 20637623 PMC2928141

[B75] Zoladz P. R. Park C. R. Diamond D. M. (2011). “Neurobiological basis of the complex effects of stress on memory and synaptic plasticity,” in The Handbook of Stress: Neuropsychological Effects on the Brain, ed. Conrad C. D. (Oxford: Wiley-Blackwell), 157–178.

[B76] Zuj D. V. Norrholm S. D. (2019). The clinical applications and practical relevance of human conditioning paradigms for posttraumatic stress disorder. Prog. Neuropsychopharmacol. Biol. Psychiatry 88, 339–351. 10.1016/j.pnpbp.2018.08.014 30134147

